# Aurora-A down-regulates IkappaBα via Akt activation and interacts with insulin-like growth factor-1 induced phosphatidylinositol 3-kinase pathway for cancer cell survival

**DOI:** 10.1186/1476-4598-8-95

**Published:** 2009-11-05

**Authors:** Jin-e Yao, Min Yan, Zhong Guan, Chao-bin Pan, Liang-ping Xia, Chuan-xing Li, Li-hui Wang, Zi-jie Long, Yan Zhao, Ming-wei Li, Fei-meng Zheng, Jie Xu, Dong-jun Lin, Quentin Liu

**Affiliations:** 1 State Key Laboratory of Oncology in South China, Cancer Center, Sun Yat-sen University, 651 Dongfeng Road East, Guangzhou, PR China; 2 Department of Otorhinolaryngology, Second Affiliated Hospital, Sun Yat-sen University, Yanjiang Road West, Guangzhou, PR China; 3 Department of Maxillofacial Surgery, Second Affiliated Hospital, Sun Yat-sen University, Yanjiang Road West, Guangzhou, PR China; 4 Department of Comprehensive, Cancer Center, Sun Yat-sen University, 651 Dongfeng Road East, Guangzhou, PR China; 5 Department of Medical Imaging & Interventional Radiology, Cancer Center, Sun Yat-sen University, 651 Dongfeng Road East, Guangzhou, PR China; 6 Department of Hematology, Third Affiliated Hospital, Sun Yat-sen University, Zhongshan Road, Guangzhou, PR China

## Abstract

**Background:**

The mitotic Aurora-A kinase exerts crucial functions in maintaining mitotic fidelity. As a bona fide oncoprotein, Aurora-A aberrant overexpression leads to oncogenic transformation. Yet, the mechanisms by which Aurora-A enhances cancer cell survival remain to be elucidated.

**Results:**

Here, we found that Aurora-A overexpression was closely correlated with clinic stage and lymph node metastasis in tongue carcinoma. Aurora-A inhibitory VX-680 suppressed proliferation, induced apoptosis and markedly reduced migration in cancer cells. We further showed that insulin-like growth factor-1, a PI3K physiological activator, reversed VX-680-decreased cell survival and motility. Conversely, wortmannin, a PI3K inhibitor, combined with VX-680 showed a synergistic effect on inducing apoptosis and suppressing migration. In addition, Aurora-A inhibition suppressed Akt activation, and VX-680-induced apoptosis was attenuated by Myr-Akt overexpression, revealing a cross-talk between Aurora-A and PI3K pathway interacting at Akt activation. Significantly, we showed that suppression of Aurora-A decreased phosphorylated Akt and was associated with increased IkappaBα expression. By contrast, Aurora-A overexpression upregulated Akt activity and downregulated IkappaBα, these changes were accompanied by nuclear translocation of nuclear factor-κB and increased expression of its target gene Bcl-xL. Lastly, Aurora-A overexpression induced IkappaBα reduction was abrogated by suppression of Akt either chemically or genetically.

**Conclusion:**

Taken together, our data established that Aurora-A, via activating Akt, stimulated nuclear factor-κB signaling pathway to promote cancer cell survival, and promised a novel combined chemotherapy targeting both Aurora-A and PI3K in cancer treatment.

## Background

Mammalian Aurora kinases, including Aurora A, B, and C, represent a new family of serine/threonine kinases crucial for several physiological processes including cytokinesis and chromosome segregation [1-3]. Aberrant expression and activity of Aurora kinase lead to formation of abnormal spindle in mitosis and aneuploidy which are closely associated with genomic instability [1,4]. Indeed, Aurora-A (Aur-A) is frequently overexpressed in various cancer types, such as ovarian, breast, colorectal, pancreatic, bladder and gastric cancer [5-7]. Overexpression of Aur-A induces tumorigenesis, metastasis and chemoresistance, correlating with its pro-survival function in cancer cells. Thus, Aurora kinase has been considered to be an oncoprotein and a promising molecular target for cancer therapy.

We and others previously reported that Aur-A-induced cell survival and migration were correlated with Akt activation [8,9]. Phosphatidylinositol 3-kinase (PI3K)/Akt signaling pathway is involved in survival and invasion in human cancers [10-12]. Akt, which consists of a family of highly conserved serine/threonine kinases, plays a key role in mediating insulin-like growth factor-1 (IGF-1)-stimulated cell survival response. Many pro-apoptotic proteins have been identified as direct or indirect Akt substrates, including glycogen synthase kinase-3 (GSK-3), Bad and forkhead transcription factors [13]. In addition, Aur-A was reported to up-regulate NF-κB signaling by phosphorylation of IkappaBα(IκBα) [14]. NF-κB stimulates proliferation and blocks apoptosis via modulating transcription of pro-survival genes such as Bcl-xL and Bcl-2 in a number of cancer cell types [15]. Intra-cellular negative regulation of NF-κB is controlled primarily through interactions with IκB family, which prevent nuclear translocation and DNA binding of NF-κB. The exact mechanism and pathway by which Aur-A promotes cancer cell survival and anti-apoptosis however remain unclear.

Tongue squamous cell carcinoma (TSCC), the common type of head and neck squamous cell carcinoma, is associated with a high mortality rate. The poor survival of tongue cancer is mainly due to tumor recurrence and regional lymph node metastasis, the most reliable prognostic indicators for patients [16]. Enhanced cytotoxicity has been observed when anti-EGFR monoclonal antibody cetuximab (Erbitux, C225) is used in combination with a number of conventional cytotoxic therapies, including cisplatin and paclitaxel to avoid the severe side-effect. Thus designing new drugs or combined chemotherapy aiming to enhance cytotoxicity and attenuate side-effect becomes urgent and challenging tasks.

In this study, we first showed that Aur-A was overexpressed in TSCC tissues and closely correlated with lymph node metastasis in patients. Aur-A inhibitory VX-680 [17,18] demonstrated a potent anti-tumor activity against various aspects of TSCC tumor progression, offering an opportunity for target therapy. More interestingly, we showed that activation of PI3K signaling by IGF-1 abrogated Aur-A inhibitory VX-680 induced apoptosis, whereas combination of VX-680 and PI3K inhibitor induced synergistic effects on inducing apoptosis and reducing migration in cancer cells. These data suggested a cross-talk between Aur-A and PI3K signaling pathway in regulating cell survival and migration. More importantly, we found that Aur-A downregulated IκBα via Akt activation, and subsequently induced NF-κB p65 translocated to nuclei where expression of its target gene Bcl-xL was increased, pointing that Aur-A promoted cell survival via Akt-mediated IκB kinase (IKK)/NF-κB signaling pathway. Taken together, understanding the mechanism underlying the pro-survival activity of Aur-A and the link between Aur-A and PI3K pathway provide a new insight and rationale for future combined molecular targeting therapeutics.

## Results

### Aur-A is overexpressed in TSCC tissues and correlated with clinical stage and lymph node metastasis

**Figure 1:**
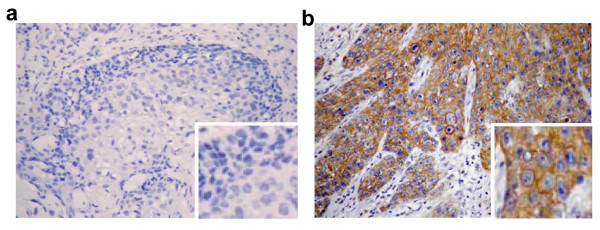
**Aur-A is overexpressed in TSCC tissues and correlated with clinical stage and lymph node metastasis**. TSCC or its corresponding adjacent normal samples were collected and subjected to immunohistochemical staining with Aur-A antibody. (a) Aur-A expression is low in normal samples. (b) Aur-A expression is obviously increased in TSCC, original magnification, × 200. Insets show enlarged views, original magnification, × 400.

We used the immunohistochemical analysis to investigate Aur-A expression in primary tumor tissues. Results showed that only a few (7/30, 23.3%) matched adjacent normal tissues displayed Aur-A positive staining (Fig. 1a). However, Aur-A was significantly elevated in majority (36/55, 65.5%) of pathologically confirmed tumor specimens (Fig. 1b). Aur-A was uniformly cytoplasmic positive staining, uncoupled with its normal mitosis-related expression pattern.

We further analyzed the relationship between Aur-A expression and clinical characteristics (Table 1). Aur-A was more frequently expressed in high-grade tumors (stage III and IV, 77.8%) compared with low-grade (stage I and II, 42.1%) tumors (*p *= 0.008). Moreover, we observed preferential expression of Aur-A in tumor with positively (87.5%) versus negatively (56.4%) lymph node metastasized samples (*p *= 0.028). No significant correlation was found between Aur-A expression and other clinical characteristics including age, gender and differentiation status. Thus, the potential association between tumor overexpression of Aur-A and clinic stage or lymph node metastasis raises the possibility of specific inhibition of Aurora kinase in treatment of tongue cancer cells.

**Table 1:** Association of Aur-A expression with clinicopathological parameters

Clinicopathological features		n	Aur-A expression Positive No.(%)	χ^2^	*P*
Age	≤ 60 years	28	20 (71.4)	0.900	0.343
	>60 years	27	16 (59.3)		
Gender	Male	33	22 (66.7)	0.054	0.817
	Female	22	14 (63.6)		
Clinical Stage	I+II	19	8 (42.1)	6.999	0.008
	III+IV	36	28 (77.8)		
Differentiation	Well	7	2 (28.6)	4.952	0.084
	Moderate	26	19 (73.1)		
	Poor	22	15 (68.2)		
Lymph node metastasis	Positive	16	14 (87.5)	4.850	0.028
	Negative	39	22 (56.4)		

### Aurora kinase inhibitory VX-680 suppresses cell growth and induces apoptosis in a dose-dependent manner in TSCC cells

**Figure 2:**
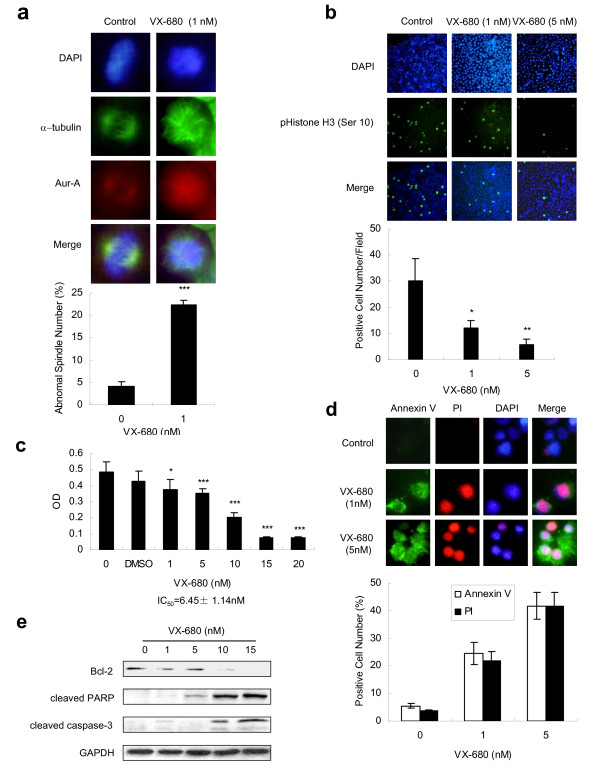
**Aurora kinase inhibitory VX-680 suppresses cell growth and induces apoptosis in TSCC cells**. Cells were maintained at DMSO (served as a control) or VX-680 for 48 h. (a and b) VX-680 inhibits Aurora kinase and leads to defects in mitotic spindles. Cells were subjected to immunofluorescence staining with α-tubulin (green), Aur-A (red, original magnification × 600) or pHistone H3-Ser10 antibodies (green, original magnification × 200). DAPI (blue) was used to visualize the nuclei. (a) Quantification showed the percentage of the abnormal spindles assessed as monopolarity of three independent experiments. In each experiment, at least 150 randomly chosen spindles were counted. (b) Histogram indicated the number of pHistone H3 positive cells counted in five randomly selected fields from three independent experiments. Error bars indicated the SD. *p < 0.05, **p < 0.01, compared to control. (c-e) VX-680 suppresses cell growth and induces apoptotic cell death. (c) Cell survival rates were measured by MTT assay, *p < 0.05, ***p < 0.001. (d) Representative immunofluorescent images of apoptotic cells were stained with Annexin V (green), PI (red) and DAPI (blue). Histogram represented the percentage of Annexin V or PI positive staining cells of three independent experiments. (e) Cell apoptosis was analyzed by Western blot with indicated antibodies. GAPDH was used as a control.

To evaluate the inhibition of Aurora kinase in TSCC cells, we used a small molecule inhibitor VX-680. Figure 2a showed that the percentage of abnormal spindle as was markedly increased in VX-680 treated mitotic cells (22.39 ± 0.98%) compared to the control mitotic cells (4.21 ± 0.91%). The abnormal spindle characterized as mono-polarity consistent with a known Aur-A inhibition phenotype [19]. Phosphorylation inhibition of histone H3 at Ser10, an *in vivo *substrate of Aur-B was significantly reduced in Tca8113 cells treated with VX-680 at 1 nM (12.00 ± 3.06) or 5 nM (5.80 ± 0.08), compared to the control cells (30.20 ± 8.62, Fig. 2b).

Cell survival rates were reduced by VX-680 in a dose-dependent manner as assessed by MTT assay with IC_50 _of 6.45 ± 1.14 nM (Fig. 2c). Annexin V assay revealed that VX-680 induced apoptosis even at 1 nM as showed in Annexin V and PI staining positive (Fig. 2d). Western blot assay showed that VX-680 reduced the expression of anti-apoptotic protein Bcl-2 and increased the level of both cleaved PARP and cleaved caspase-3 in a dose-dependent manner (Fig. 2e). Caspase-3 inhibitor however reversed Bcl-2 reduction and PARP cleavage in response to VX-680 (data not shown).

### Cross-talk between Aur-A and PI3K pathway regulates VX-680 induced apoptosis in tumor cells

**Figure 3:**
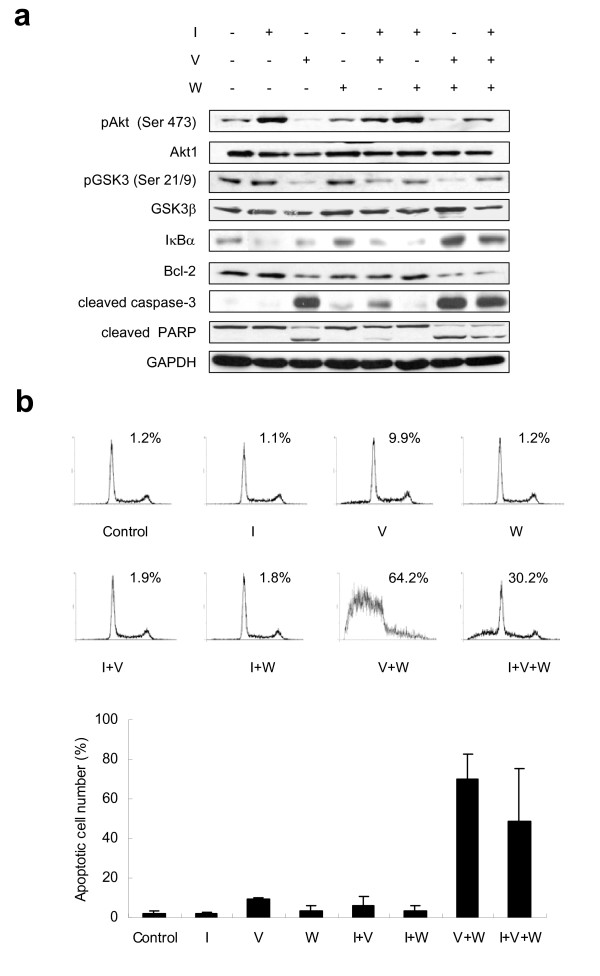
**Cross-talk between Aur-A and PI3K pathway regulates VX-680 induced apoptosis in TSCC cells**. Serum-starved Tca8113 cells treated with IGF-1 (I, 100 ng/ml), wortmannin (W, 1 μM), VX-680 (V, 5 nM) alone or in combination for 12 h. (a) Cells were subjected to Western blot analysis with indicated antibodies. GAPDH was used as a control. (b) The apoptosis was assessed by flow cytometry as a sub-G1 population. Images showed one representative of three independent experiments. Histogram represented the quantification.

Using a serum-free system, we examined cell apoptosis by Western blot and flow cytometry assay. IGF-1 increased the phosphorylation of Akt at Ser473 and its downstream target GSK3 at Ser 21/9. Expression of IκBα was however decreased by IGF-1 treatment (Fig. 3a), which also prevented VX-680 (5 nM)-induced apoptosis (Fig. 3b). Interestingly, VX-680 and an irreversible PI3K inhibitor wortmannin in combination displayed a dramatic effect in inhibiting Akt and GSK3 activity, elevating IκBα expression (Fig. 3a), and increasing cell apoptosis, compared with either VX-680 (about 7.90 ± 2.54-fold) or wortmannin (about 18.49 ± 2.88-fold) alone (Fig. 3b). We calculated the cooperative coefficient of VX-680 and wortmannin was 6.09 ± 0.35, suggesting wortmannin synergized VX-680 mediated apoptosis by inhibiting PI3K. Meanwhile, elevated levels of cleaved PARP and cleaved caspase-3 and reduction of Bcl-2 expression were observed in cells treated with VX-680 and/or wortmannin (Fig. 3a). These data together indicated that there was an intracellular cross-talk between Aurora kinases and PI3K pathway in regulating cancer cell survival. We conducted Western blot with another squamous carcinoma KB cells and observed similar results (Additional file 1).

### Aur-A interacts with PI3K pathway in regulating TSCC cell migration

We have showed that overexpression of Aur-A was positively correlated with lymph node metastasis (Table 1), and cell migration was closely associated with potential of tumor invasiveness and metastasis. We showed that VX-680 potently induced a dose-dependent inhibition in the migration of Tca8113 cells (Additional file 2). Similar inhibition of cell motility was also induced by Akt/protein kinase B signaling inhibitor-2 (API-2) at dose of 1 μM.

**Figure 4:**
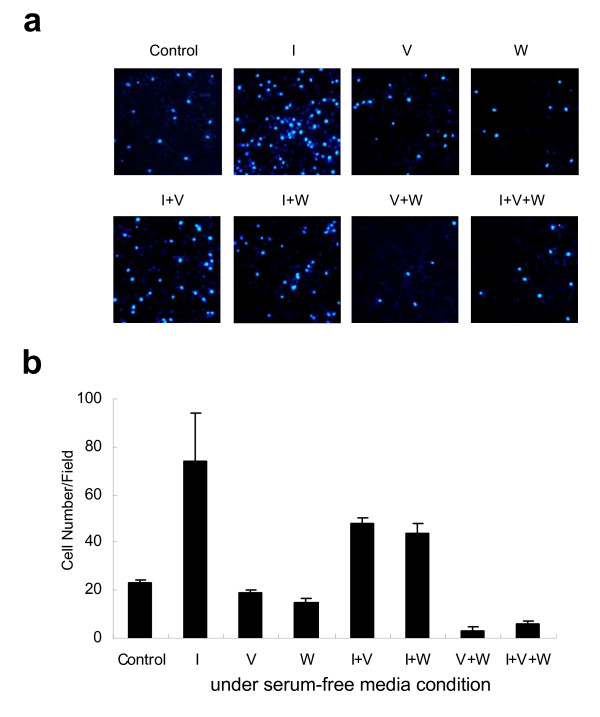
**Aur-A interacts with PI3K pathway in regulating TSCC cell migration**. Cells were incubated in serum-free media containing IGF-1 (I, 100 ng/ml), wortmannin (W, 1 μM), VX-680 (V, 1 nM) alone or in combination for 16 h. Migration rates were quantified by counting the migrated cells in five random fields. (a) One representative of three independent experiments was shown, original magnification × 200. (b) Data summarized three independent experiments in identical condition.

We then conducted the transwell migration assay in serum-free condition. Compared with the control cells, IGF-1 significantly enhanced migration of Tca8113 cells (about 3.5-fold), while either VX-680 or wortmannin alone at low dose could partially reduce the cell mobility induced by IGF-1 (Fig. 4). Moreover, the combination of VX-680 and wortmannin efficiently abrogated IGF-1 induced cell migration in a synergic manner. Meanwhile we performed MTT assay to detect the cell viability in the same system. These results showed that the suppression of migration by VX-680 and/or wortmannin were not due to inducing apoptosis in Tca8113 cells (data not shown). Thus, these data indicated the interaction between Aurora kinases and PI3K pathway also played a key role in cancer cell migration.

### Activated Akt attenuates Aur-A inhibitory VX-680-induced apoptosis in TSCC cells

**Figure 5:**
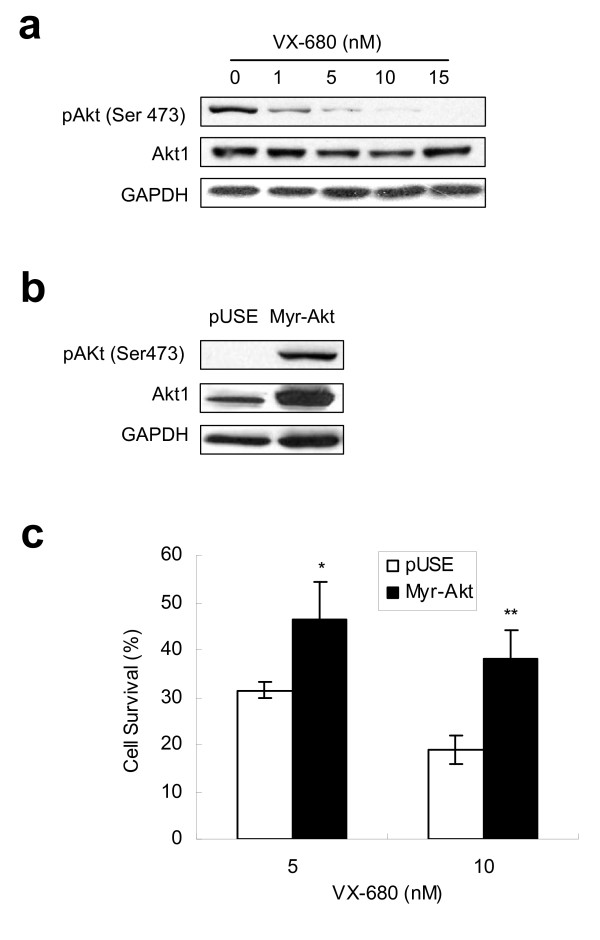
**Activated Akt attenuates Aur-A inhibitory VX-680-induced apoptosis in TSCC cells**. (a) Cells were incubated in serum-free media with indicated doses of VX-680 for 24 h, and subjected to Western blot analysis with pAkt (ser473), and Akt1 antibodies. (b) Myr-Akt1 or pUSE stable transfected cells were subjected to Western blot with pAkt and Akt1 antibodies, GAPDH was used as a control. (c) Myr-Akt1 or pUSE transfected cells were treated with VX-680 (5 nM or 10 nM) for 24 h. Cell survival rates were measured by MTT assay.

Based on above findings, we hypothesized that Aur-A and PI3K pathway might interact at Akt. The level of pAkt was decreased in cells treated with increasing concentration of VX-680 (Fig. 5a). We further overexpressed a constitutively active form of Akt (Myr-Akt1) in Tca8113 cells (Fig. 5b). MTT assay showed that the survival rate of Myr-Akt1 transfected cells was (46.43 ± 7.95% and 38.11 ± 6.16%), obviously higher than that of empty vector pUSE transfected cells (31.5 ± 1.67% and 18.93 ± 2.90%) when treated with VX-680 at 5 nM and 10 nM respectively (Fig. 5c). We performed Aur-A RNAi in vector or Myr-Akt1 transfected cells and observed similar results (Additional file 3). Together, these data suggested that Akt was a potential downstream target of Aurora kinases in enhancing cancer cell survival.

### Aur-A down-regulates IκBα via Akt phosphorylation and induces p65 subunit of NF-κB nuclear translocation

**Figure 6:**
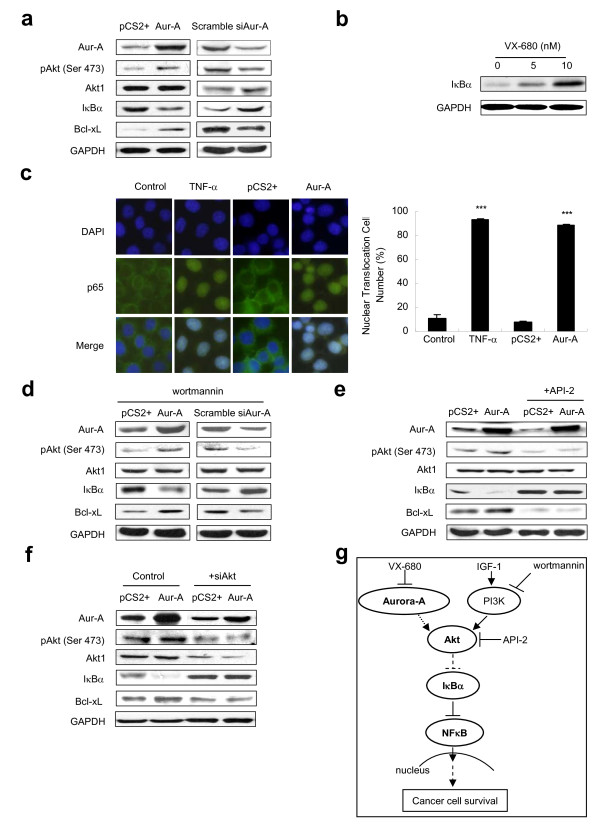
**Aur-A down-regulates IκBα via Akt phosphorylation and induces p65 subunit of NF-κB nuclear translocation**. (a) Cells were transiently transfected with Aur-A or pCS2+, or Aur-A siRNA or its scramble control. (b) Cells were incubated in serum-free media with VX-680 for 12 h. (c) Cells were treated with or without TNF-α 50 ng/ml, or transiently transfected with Aur-A or pCS2+, and subjected to immunofluorescence staining with anti-p65 antibody (green). DAPI (blue) was used to visualize the nuclei, original magnification × 1000. Histogram represented the percentage of cells with nuclear translocation from three independent experiments. (d) Aur-A or pCS2+, Aur-A siRNA or its control transfected cells treated with wortmannin 1 μM for 24 h before harvesting. (e) Cells were transfected with Aur-A or pCS2+ and treated with API-2 1 μM for 24 h prior to harvesting. (f) Cells were cotransfected Aur-A or pCS2+ with Akt1 siRNA or its control. Cell lysates were analyzed for indicated proteins by Western blot (a, b and d-f). (g) A diagram depicts the Aur-A-mediated pro-survival signaling pathways.

A recent study reported that Aur-A regulated NF-κB via phosphrylation of IκBα [14]. We further studied whether Aur-A regulated IκBα and its downstream targets via Akt pathway. Decreased pAkt and elevated IκBα were detected when cells were transfected with siRNA toward either Akt (Additional file 4) or Aur-A (Fig. 6a), compared with cells transfected with their scramble control respectively. Inhibition of Aur-A chemically also up-regulated IκBα level (Fig. 6b). Conversely, overexpression of Aur-A increased Akt activity and decreased IκBα level compared with the vector control (Fig. 6a). We then analyzed the expression of Bcl-xL, which is known as a NF-κB target gene closely associated with cell proliferation and apoptosis. Bcl-xL was down-regulated in Aur-A and Akt depleted cells (Fig. 6a). Immunofluorescence staining of NF-κB p65 showed that Aur-A overexpression was significantly associated with p65 nuclear translocation whereas p65 was mainly expressed in the cytoplasm in cells transfected with empty vector pCS2+ (Fig. 6c). We further showed that inhibition of PI3K with wortmannin did not prevent either an increase of pAkt and Bcl-xL or a decrease in IκBα caused by Aur-A overexpression (Fig. 6d). Interestingly, in cells incubated with Akt inhibitor API-2 or siRNA against Akt, overexpression of Aur-A however failed to reduce IκBα or raise Bcl-xL expression in comparison to the vector control (Fig. 6e and 6f). This suggested that Akt, but not PI3K, was involved in the down-regulation of IκBα by Aur-A. These results revealed that Aur-A, via its downstream target Akt, down-regulated IκBα, which then led to NF-κB nuclear translocation and subsequently activating NF-κB target gene Bcl-xL in enhancing cancer cell survival (Fig. 6g).

## Discussion

Aur-A kinase plays a critical role in tumorigenesis as an oncogenic protein. However, the exact pathway by which Aur-A enhances cell survival has not been well defined. In this study, we showed that Aur-A, via activating Akt pathway, induced NF-κB nuclear translocation to promote cell survival. Indeed, overexpression of Aur-A was positively associated with clinic stage and lymph node metastasis in TSCC patients. Moreover, we established a cross-talk between mitotic Aurora kinase and IGF-1-induced PI3K survival pathway, interacting at Akt activation. Combined inhibition of both Aur-A and PI3K led to a synergistic effect on inducing apoptosis and suppressing migration, reassuring an emerging theme of combination therapy in cancer treatment.

Aur-A, a key regulator of mitosis, is essential for centrosome function, spindle assembly, and mitotic entry [1-3]. Dysregulation of Aur-A has been linked to tumorigenesis. Previous studies have also shown that Aur-A functions as a pro-survival protein that counteract apoptosis and induce drug resistance in tumour cells [20]. We and others demonstrated that Aur-A promoted cell survival and migration by Akt activation, and Aur-A activated NF-κB via IκBα phosphorylation [8,9,14]. Nevertheless, a clear pathway from Aur-A activation to cell survival remains to be elucidated. In this study, we showed that inhibition of Aur-A induced cell apoptosis accompanied with suppressing Akt activation, increasing IκBα level and down-regulating Bcl-xL expression. On the contrary, overexpression of Aur-A led to Akt activation and IκBα down-regulation, subsequently induced NF-κB p65 nuclear translocation to enhance cell survival. Moreover, suppression of Akt by either API-2 or siAkt prevented Aur-A-induced IκBα reduction and Bcl-xL elevation. Thus, our data demonstrated that Aur-A downregulated IκBα via Akt activation, triggering NF-κB p65 nuclear translocation, and subsequently activating target gene Bcl-xL to promote survival in cancer cells.

Inactivation of PTEN leads to constitutively activate PI3K/Akt pathway. Recently, Aur-A was found to abrogate the DNA-binding and transactivation activity of p53 and subsequently inhibit its downstream target genes including *PTEN *by phosphorylating Ser 215 [21]. PTEN expression was significantly reduced in Aur-A overexpressed cells with activated Akt activity [22]. Here, we showed that overexpression of Aur-A increased the phosphorylation of Akt at Ser 473 (Fig. 6a). Consistently, previous report showed that Aur-A activated Akt in a p53-dependent manner to induce cell survival and chemoresistance in ovarian cancer cells [9]. Thus, it is conceivable that Aur-A activates Akt via inhibiting PTEN.

Akt promotes cell survival by its ability to phosphorylate and inactivate several pro-apoptotic targets including GSK-3. We showed that inhibition of Aur-A resulted in suppressed phosphorylation of both Akt and GSK-3, according with one recent study that Aur-A promoted cell proliferation by increasing the phosphorylation of GSK-3β [23]. On the other hand, another work reported that Akt inhibitor A-443654 interfered with mitotic progression by decreasing Aur-A expression, suggesting Akt acts upstream of Aur-A by regulating its transcription level [24]. We and others showed that Aur-A contributed to cell survival, chemoresistance and migration via activation of Akt, suggesting a positive feedback interplaying between Aur-A and Akt.

Akt plays a part in activation of NF-κB signaling pathway and exerts a positive effect on NF-κB function by phosphorylation and activation of IKK, a kinase that phosphorylates and induces proteolytic degradation of the NF-κB inhibitor, IκBα [25]. Interestingly, several recent reports have suggested that Aur-A kinase may serve both upstream and downstream of the IKK complex components [14,26]. IKK complex includes two catalytic components, IKKα and IKKβ. As a downstream target, Aur-A was phosphorylated by IKKα at threonine residue 288, a site which is important for its kinase activity [26]. Depletion of IKKβ resulted in the up-regulation of Aur-A protein, and IKKβ functioned as an antagonist of Aur-A signaling during mitosis in normal cells [27]. On the other hand, we showed that Aur-A promoted cell survival through activated IKK/NF-κB signaling pathway, consistent with previous reports [14,28]. Thus, there may be a reciprocal regulation between Aur-A and IKK complex.

Activation of Akt was associated with adverse outcome in tongue cancer patients, serving as a significant prognostic factor in TSCC [29]. Multiple growth factors such as IGF-1, VEGF, and EGF facilitate the development and progression of cancer by activating PI3K pathway leading to cell survival and therapeutic resistance [30-32]. Here, we showed that Aur-A was overexpressed in tongue cancer tissue and tightly correlated with clinical stage and lymph node metastasis in patients (Fig. 1 and Table 1). Thus, dysregulation of mitotic Aur-A kinase and abnormal activation PI3K survival pathway are two essential but distinct biological processes in cancer progression. As tumorigenesis is a multiple process, combination therapeutic strategies have shown substantially enhanced anti-tumor effects and reduced side-effects both *in vitro *and *in vivo*. A recent study reported that combined treatment with the pan-histone deacetylase inhibitor vorinostat and Aur-A kinase inhibitor MK-0457 (VX-680) showed a synergistic anti-leukemia activity in cultured and primary AML and CML cells [33]. Here, we demonstrated that Aur-A inhibitory VX-680 could markedly reduce IGF-1 induced survival and migration. Furthermore, combinational inhibition of Aur-A and PI3K showed a synergic effect in causing apoptosis and suppressing migration in cancer cells (Fig. 3, 4 and Additional file 1).

## Conclusion

Taken together, our findings demonstrated that Aur-A stimulated NF-κB signaling pathway via Akt activation to promote cancer cell survival, and formed a conceptual basis for the combination chemotherapy of targeting both Aurora kinase and growth factor-induced PI3K pathway for inhibiting the enhanced survival and migration of cancer cells.

## Methods

### Patients and clinical tissue specimens

Fifty-five patients who performed radical surgery were original clinically diagnosed and pathologically confirmed of TSCC between 1987 and 1992. Pertinent patient clinical reports were obtained with prior patient consent and the approval of the institutional Clinical Ethics Review Board. All of the 55 specimens and additional 30 normal adjacent tissues were collected and fixed in formalin and embedded in paraffin in the diagnostic histopathology laboratory at the Second Affiliated Hospital of Sun Yat-sen University. Patient clinic pathological features were shown in Table 1. Tumors were staged according to UICC classification (1997): stage I (4 cases), II (15 cases), III (23 cases) and IV (13 cases) or histopathology classification: stage I (7 cases) stage II (26 cases) and stage III (22 cases).

### Reagents and cell lines

VX-680 was purchased from Kava Technology, San Diego, CA., API-2 was from Calbiochem, IGF-1 from Biosource, tumor necrosis factor α (TNF-α) and wortmannin from Cell Signaling. Human tongue squamous cancer cell line Tca8113 was kindly provided by Xiao-feng Zhu (Cancer Center, Sun Yat-sen University), human oral floor cancer cell line KB was obtained from ATCC.

### Immunohistochemical staining of Aur-A expression

Aur-A immunohistostaining using an anti-Aur-A antibody (Upstate) on tongue cancer tissues was performed as previously described [8]. Moderate or strong cytoplasm staining, considered as positive reaction, was assessed semi-quantitatively by at least two independent pathologists. Specimen was determined as positive staining for Aur-A when >30% cells showed visible brown granules in the cytoplasm.

### Immunofluorescence staining

Cultured cells grown on coverslips treated with DMSO or VX-680, or transiently transfected with plasmid expressing Aur-A or empty vector pCS2+. Immunofluorescence staining of cells was performed as described [34] and analyzed with an Olympus BX51 microscope. For immunofluorescence staining of NF-κB p65, cells were treated with 50 ng/ml of TNF-α for 10 min prior to fixing as a positive control.

### MTT assay

Tca8113 cells were incubated in 96-well plate and maintained at different doses of VX-680 for 48 h. Myr-Akt or pUSE transfected Tca8113 cells were maintained at different doses of VX-680 for 24 h. Cell survival was assessed as described previously [35].

### Flow cytometry analysis

Cells were incubated in serum-free media with indicated drugs for 12 h and subjected to flow cytometry analysis as previously described [34].

### Annexin V assay

Cells were treated with DMSO or VX-680 for 48 h prior to collecting and resuspending in binding buffer. Annexin V-FITC and propidium iodide (Annexin V-FITC Apoptosis Detection Kit, Merck) were added to each sample according to the manufacturer's protocol. 4, 6-diamidino-2-phenylindole (DAPI 1 μg/ml) was used to visualize nuclei. 20~25 μl of cell suspension was transfered onto glass microscope slides respectively, and viewed immediately using a fluorescence microscope (Olympus BX51).

### Western blot assay

Western blot assay was performed as described previously [8]. Antibodies used were mouse anti-GAPDH (Ambion), rabbit anti-Bcl-2, rabbit anti-cleaved caspase-3, mouse anti-cleaved PARP (Asp175), rabbit anti-phosphorylated Akt (pAkt, Ser473), mouse anti-phospho-GSK3α/β (Ser 21/9, Cell Signaling), mouse anti-IκBα (BD), rabbit anti-GSK3β, goat anti-Akt1, rabbit anti-Bcl-xL (Santa Cruz Biotechnology) and rabbit anti-Aur-A (Upstate).

### Generation of stable transfection cell lines

Myr-Akt1 and pUSE plasmids were generously provided by Xiao-feng Zhu (Cancer Center, Sun Yat-sen University). Transfections were conducted according to manufacturers' recommendations (Invitrogen). Tca8113 cell clones stably transfected with plasmid were selected in 400 μg/ml G418.

### Transient transfection and cotransfection

Transient transfection of Aur-A and its vector control pCS2+ or cotransfection of Aur-A or pCS2+ with siRNA against Akt1 or its control were conducted according to manufacturers' recommendations (Invitrogen). Lysates were prepared 48 h after transfection. Cells were treated with API (10 μM) or wortmannin (1 μM) for 24 h prior to collecting for Western blot.

### RNA-mediated interference

siRNA for downregulating Aur-A or Akt1 expression was done by the transfection of RNA oligonucleotides with lipofectamine 2000. The sequence for siRNA against Aur-A was AUGCCCUGUCUUACUGUCA and siRNA against Akt1 was AAGGAGGGUUGGCUGCACAAA. Lysates were prepared 36 h after transfection.

### Transwell migration assay

Transwell assay was performed as described previously [8]. Briefly, cells were incubated in serum or serum-free media containing desired drugs for 16 h. The migrated cells in five fields were counted, and the average of each chamber was determined.

### Statistics

Statistical analysis was performed using SPSS version 13.0 (SPSS Inc., Chicago, IL, USA). The χ^2 ^test and Student's t-test was used to make a statistical comparison between groups. P < 0.05 was considered statistically significant. We performed each study at least three times under identical conditions.

## Abbreviations

The abbreviations used are: PI3K: phosphatidylinositol 3-kinase; Aur-A: Aurora-A; TSCC: tongue squamous cell carcinoma; IGF-1: insulin-like growth factor-1; API-2: Akt/protein kinase B signaling inhibitor-2; GSK-3: glycogen synthase kinase-3; IKK: IκB kinase; IκBα: IkappaBα; NF-κB: nuclear factor-κB.

## Competing interests

The authors declare that they have no competing interests.

## Authors' contributions

JY carried out protein studies, apoptosis analysis, transwell assays, immunofluorescence staining, statistical analysis and co-wrote the manuscript. MY drafted the manuscript, contributed to the study design and participated in the performed the protein studies, apoptosis analysis, transwell assays, immunofluorescence staining, statistical analysis. ZG collected the samples and reviewed specimen pathology. CB P participated in sample collection. LP X contributed to the sample acquisition and study design. CX L contributed to reviewed specimen pathology and manuscript editing. LH W performed gene transduction and edited the manuscript. ZJ L participated in the apoptosis assay and transwell assay. MW L contributed to the sample acquisition and statistical analysis. YZ participated in immunofluorescence staining. FM Z participated in gene transduction and edited the manuscript. JX contributed to reviewed specimen pathology and the protein expression studies. DJ L participated in the design of the study and the statistical analysis. QL conceived of the study, and participated in its design and coordination and helped to draft the manuscript. All authors read and approved the final manuscript.

## Supplementary Material

Additional file 1 **Cross-talk of Aur-A and PI3K pathway regulates VX-680-induced apoptosis in KB cells**. Serum-starved KB cells treated with IGF-1 (I, 100 ng/ml), wortmannin (W, 1 μM), VX-680 (V, 2 nM) alone or in combination for 12 h. Cells were subjected to Western blot analysis with indicated antibodies. GAPDH served as a loading control. Click here for file

Additional file 2 **VX-680 suppresses Tca8113 cell migration**. Cells were incubated in media containing 10% FBS with API-2 1 μM or increased dose of VX-680 for 16 h. Migration rates were quantified by counting the migrated cells in five random fields. (a) One representative of three independent experiments was shown, original magnification ×200. (b) Data summarized three independent experiments, *p < 0.05, **p < 0.01. Click here for file

Additional file 3 **Activated Akt overrides siAur-A induced cell death in TSCC cells**. Myr-Akt1 or pUSE stable transfected cells were transfected with Aur-A siRNA or its scramble control. Cell survival rate was determined by MTT assay. Click here for file

Additional file 4 **Downregulation of Akt increases IκBα level in TSCC cells**. Cells were transiently transfected with Akt1 siRNA or its scramble control. Cell lysates were analyzed for indicated proteins by Western blot. GAPDH was used as a control. Click here for file
