# Supplementary figures and images for: Aurora-A down-regulates IkappaBα via Akt activation and interacts with insulin-like growth factor-1 induced phosphatidylinositol 3-kinase pathway for cancer cell survival
Source: Mol Cancer. 2009 Nov 5;8:95. doi: 10.1186/1476-4598-8-95 (PMC2780390; doi:10.1186/1476-4598-8-95)

|   |   |   |   |   |   |   |   |   |
|---|---|---|---|---|---|---|---|---|
| I | - | + | - | - | + | + | - | + |
| V | - | - | + | - | + | - | + | + |
| W | - | - | - | + | - | + | + | + |

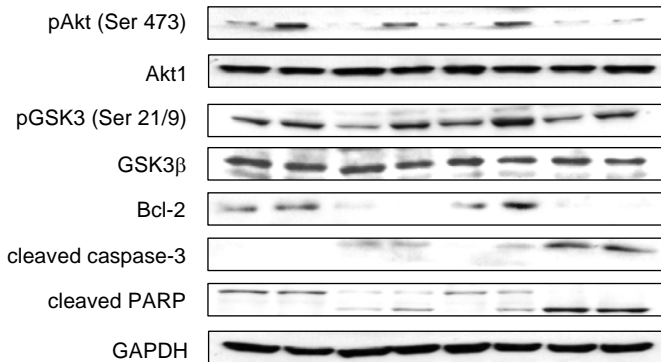

Additional file 1

Supplement: Additional file 1 — Cross-talk of Aur-A and PI3K pathway regulates VX-680-induced apoptosis in KB cells. Serum-starved KB cells treated with IGF-1 (I, 100 ng/ml), wortmannin (W, 1 μM), VX-680 (V, 2 nM) alone or in combination for 12 h. Cells were subjected to Western blot analysis with indicated antibodies. GAPDH served as a loading control. [file 1476-4598-8-95-S1.pdf]

**a**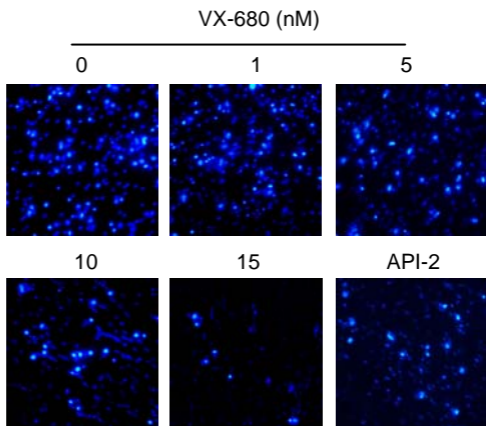**b**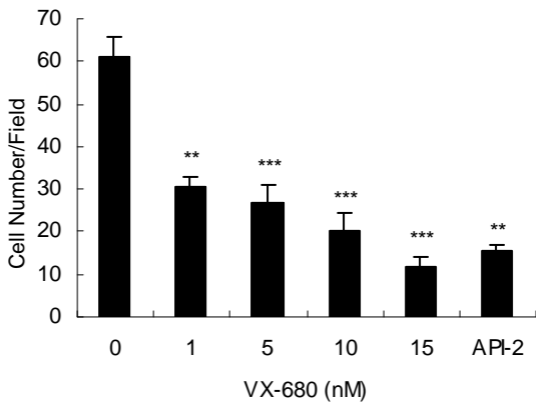

Additional file 2

Supplement: Additional file 2 — VX-680 suppresses Tca8113 cell migration. Cells were incubated in media containing 10% FBS with API-2 1 μM or increased dose of VX-680 for 16 h. Migration rates were quantified by counting the migrated cells in five random fields. (a) One representative of three independent experiments was shown, original magnification ×200. (b) Data summarized three independent experiments, *p < 0.05, **p < 0.01. [file 1476-4598-8-95-S2.pdf]

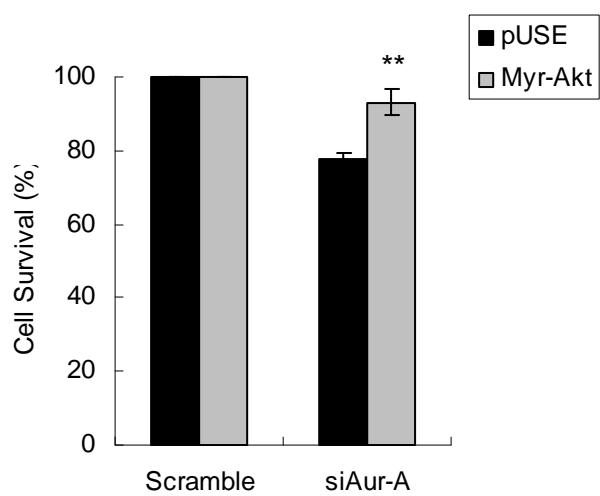

Additional file 3

Supplement: Additional file 3 — Activated Akt overrides siAur-A induced cell death in TSCC cells. Myr-Akt1 or pUSE stable transfected cells were transfected with Aur-A siRNA or its scramble control. Cell survival rate was determined by MTT assay. [file 1476-4598-8-95-S3.pdf]

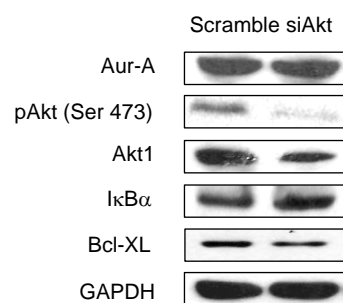

Additional file 4

Supplement: Additional file 4 — Downregulation of Akt increases IκBα level in TSCC cells. Cells were transiently transfected with Akt1 siRNA or its scramble control. Cell lysates were analyzed for indicated proteins by Western blot. GAPDH was used as a control. [file 1476-4598-8-95-S4.pdf]
